# A Study of the Preparation and Properties of Antioxidative Copper Inks with High Electrical Conductivity

**DOI:** 10.1186/s11671-015-1069-y

**Published:** 2015-09-15

**Authors:** Chia-Yang Tsai, Wei-Chen Chang, Guan-Lin Chen, Cheng-Huan Chung, Jun-Xiang Liang, Wei-Yang Ma, Tsun-Neng Yang

**Affiliations:** Institute of Nuclear Energy Research, Atomic Energy Council, Executive Yuan, Taoyuan, 32546 Taiwan

**Keywords:** Conductive ink, Stability, Resistivity, Antioxidant

## Abstract

Conductive ink using copper nanoparticles has attracted much attention in the printed electronics industry because of its low cost and high electrical conductivity. However, the problem of easy oxidation under heat and humidity conditions for copper material limits the wide applications. In this study, antioxidative copper inks were prepared by dispersing the nanoparticles in the solution, and then conductive copper films can be obtained after calcining the copper ink at 250 °C in nitrogen atmosphere for 30 min. A low sheet resistance of 47.6 mΩ/□ for the copper film was measured by using the four-point probe method. Importantly, we experimentally demonstrate that the electrical conductivity of copper films can be improved by increasing the calcination temperature. In addition, these highly conductive copper films can be placed in an atmospheric environment for more than 6 months without the oxidation phenomenon, which was verified by energy-dispersive X-ray spectroscopy (EDS). These observations strongly show that our conductive copper ink features high antioxidant properties and long-term stability and has a great potential for many printed electronics applications, such as flexible display systems, sensors, photovoltaic cells, and radio frequency identification.

## Background

In the past few years, conductive inks have attracted considerable attention due to their growing application in electrodes of silicon-crystal solar cells [1] and the printed electronics industry, such as smart labels [2], flexible displays [3, 4], and radio frequency identification (RFID) [5, 6]. Currently, silver inks have been commonly developed to enable outstanding conductivity and excellent printability [7, 8]. However, the high price and scarcity of such material limit wide industrial applications [9]. In addition, the low dispersion stability of silver ink would cause particles to aggregate, which could lower the quality of the silver thin film easily. In view of these, copper is a good alternative material for silver because of its high electrical conductivity and low price. These advantages could be highly beneficial for the reduction of the manufacturing cost. Nevertheless, a fundamental problem with copper material is its high susceptibility to oxidation in an atmospheric environment [10, 11]. Thus, the prevention of oxidation and high stability for copper inks are crucial issues to prepare conductive copper ink.

For the fabrication method of copper nanoparticles, hydrazine reductants are mostly adopted in early times [12]. Unfortunately, this process is highly toxic and dangerous, which could cause serious pollution and put people at risk of exposure when the samples are produced. However, if sodium borohydride [13] or sodium hydrophosphate [14] is utilized as the reductant, impure substances would be difficult to purify, or the synthesis needs to be performed in a vacuum environment, which leads to the cost increment. Therefore, various novel methods for synthesizing copper nanoparticles are gradually developed. For instance, copper hydroxide and l-ascorbic acid are used as the precursor salt and the reductant, respectively [15]. Such wet chemical reduction method has the advantage of avoiding toxic materials. In addition, the oxidation of products could be further prevented by using polymeric protectors. For the preparation method of conductive copper ink, a specific solvent and dispersant are very critical to disperse copper nanoparticles uniformly in the solvent and prevent aggregation of copper nanoparticles, which would influence the electrical conductivity and antioxidative ability of the copper nanoparticles. If the copper ink is oxidized easily, the quality of the silicon solar cells and the printable electronic materials would be inferior. Even if oxidation occurs after the electrodes are formed, the rapid increase in the resistance of the electrodes would reduce severely the power-generating efficiency of the silicon solar cells and affect the electrical conductivity of the printable electronic materials such as the printed circuit board (PCB). Thus, it is required to provide a method for preparing conductive copper ink having a high oxidation resistance and superior dispersibility. In this work, we present the experimental method for preparing the conductive copper ink with high antioxidant properties and investigate the electrical performances of copper ink with different solvent proportions and calcination parameters. In particular, a very high antioxidative stability for our conductive copper ink is observed. This work can greatly facilitate our abilities to develop antioxidative copper inks applied in many practical applications. For instance, we can apply quickly these copper inks to repair PCB interconnect defects occurring in the manufacture of PCBs. Furthermore, copper ink is a good candidate to substitute for silver ink especially for application to electrodes of solar cells. Interestingly, copper ink is applicable to writing. We can use a roller pen filled with copper ink to design a series of copper patterns, such as lines, electrodes, and RFID antennas. This is an easy and promising fabrication process to make conductive patterns for portable applications where the pattern is required.

## Methods

### Synthesis of Antioxidant Copper Nanoparticles

In this study, we have developed a simple method that features the all-solution processes in a non-vacuum environment and is low-pollution, low-cost, and less time-consuming (<1 h) for the synthesis of antioxidant copper nanoparticles so far. Antioxidant copper nanoparticles were synthesized using the wet chemical reduction method. Cu(OH)_2_ and PVP were dissolved in ethylene glycol solution. The solution was stirred with a magnetic stirrer for 30 min to ensure that the Cu(OH)_2_ and PVP were dissolved completely. Then, an ascorbic acid polyol solution was dissolved under the same conditions. The latter solution was poured into the former flask, and the color of the solution turned from blue to brown within 5–10 min, indicating the formation of copper nanoparticles. Finally, the resulting dispersion was washed with ethanol at 5000 rpm for 5 min via centrifugation.

### Preparation of Antioxidative Conductive Copper Ink

**Fig. 1:**
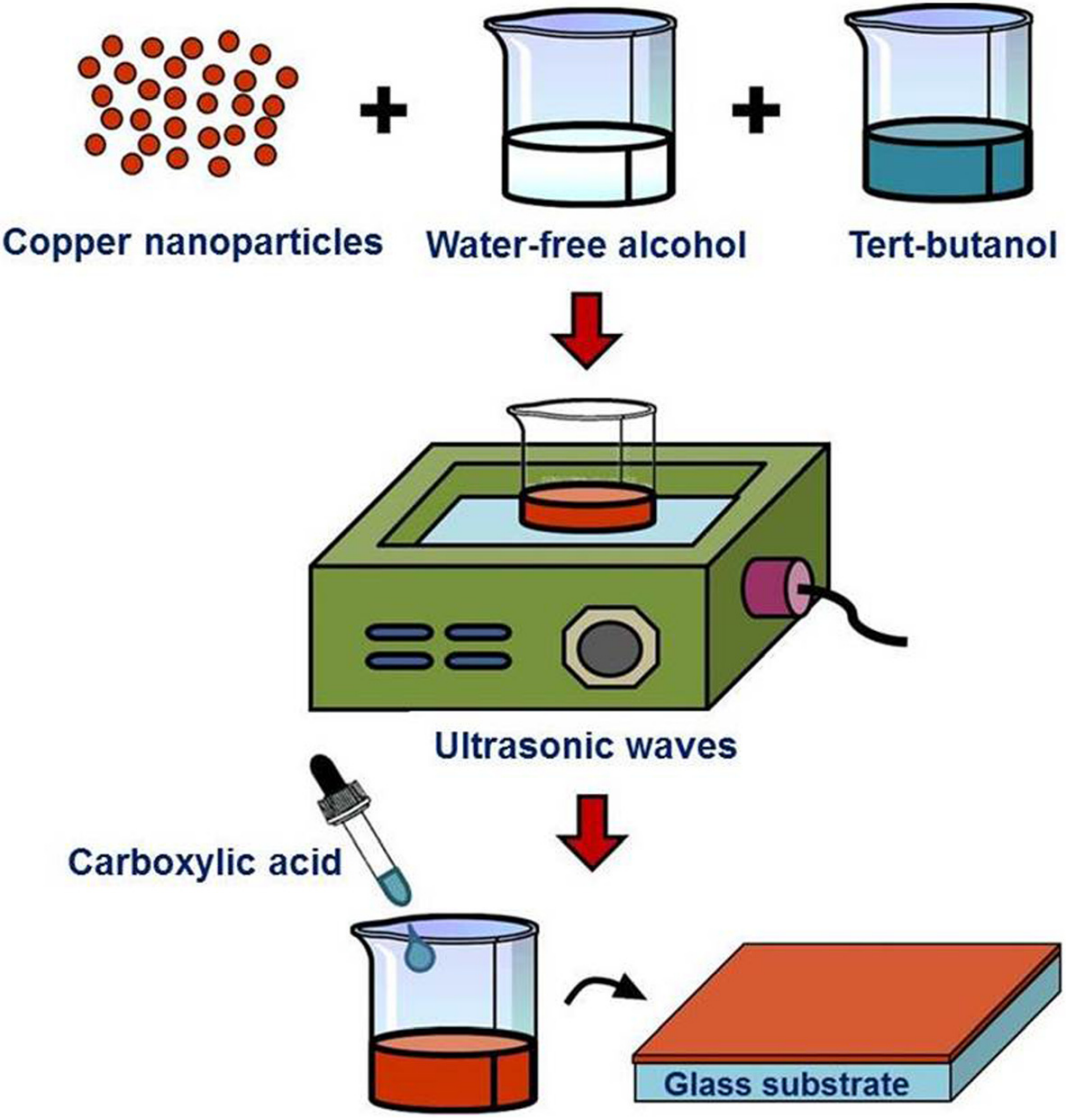
Schematic diagram of the fabrication process for preparing antioxidative conductive copper ink

Fabrication of metal nanoparticles has become one of the important topics in nanotechnology [16, 17]. For our copper nanoparticles, the wet chemical reduction method is used [18–21]. We are able to control the particle size, shape, and size distribution of copper nanoparticles. To prepare the conductive copper ink, we mix copper nanoparticles, the solvent, and the pasting agent to form a mixed solution (Fig. 1). And then, a trace of carboxylic acid is added to the mixed solution for forming the antioxidative conductive copper ink after oscillating the mixed solution using ultrasonic waves. Carboxylic acid is a solution containing a carboxyl group. Such solution can act as the dispersant to prevent aggregation of the copper nanoparticles efficiently. In this fabrication method, water-free alcohol and tert-butanol are used as the solvent and pasting agent, respectively. The composition of this mixed solution comprises a fixed weight of conductive copper nanoparticles (1 g) and different solvent proportions of water-free alcohol and tert-butanol. The ratio of the weight percentage of copper nanoparticles to that of the mixed solvent is 1:2. The solvent adopted in this study is water-free alcohol with purity higher than 99.5 %. The benefit of using water-free alcohol is to reduce the water content in the solvent as much as possible for preventing the formation of copper oxide after mixing the conductive particle material. However, the viscosity of water-free alcohol is very low. This results in the ink spraying diffusion as users dispose it on the surface of the target using printing, coating, and injecting methods. Consequently, tert-butanol is used as the pasting agent for improving the viscosity of the ink. In addition, tert-butanol would be resolved in alcohol with a boiling point of approximately 82.4 °C. Hence, it can be vaporized at a temperature lower than 100 °C, which endows it with the property of low-temperature calcination. Lastly, the finished conductive copper ink would be coated on a glass substrate using the doctor-blade method. The thickness of the spin-coated copper film is around 8 μm.

### Characterization and Measurements

The morphologies and structures of the copper nanoparticles and conductive films were characterized by field-emission scanning electron microscopy (FE-SEM, JSM-7100F). In addition, we inspected the elemental composition and distribution of conductive copper films through energy-dispersive X-ray spectroscopy (EDS) analysis by using the Oxford X-Max8 equipped in the FE-SEM. For electrical characteristics of the conductive film, we utilized the Napson RT-7 four-point probe resistance measurement system to measure sheet resistance.

## Results and Discussion

### Solvent Proportion

**Fig. 2:**
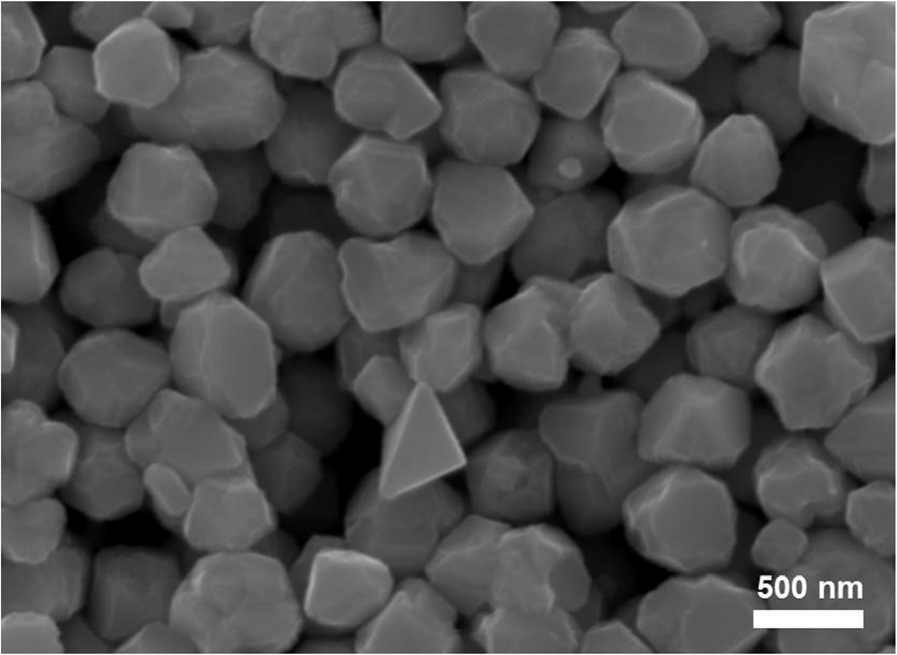
SEM image of fabricated copper nanoparticles with an average diameter of 400~500 nm

**Fig. 3:**
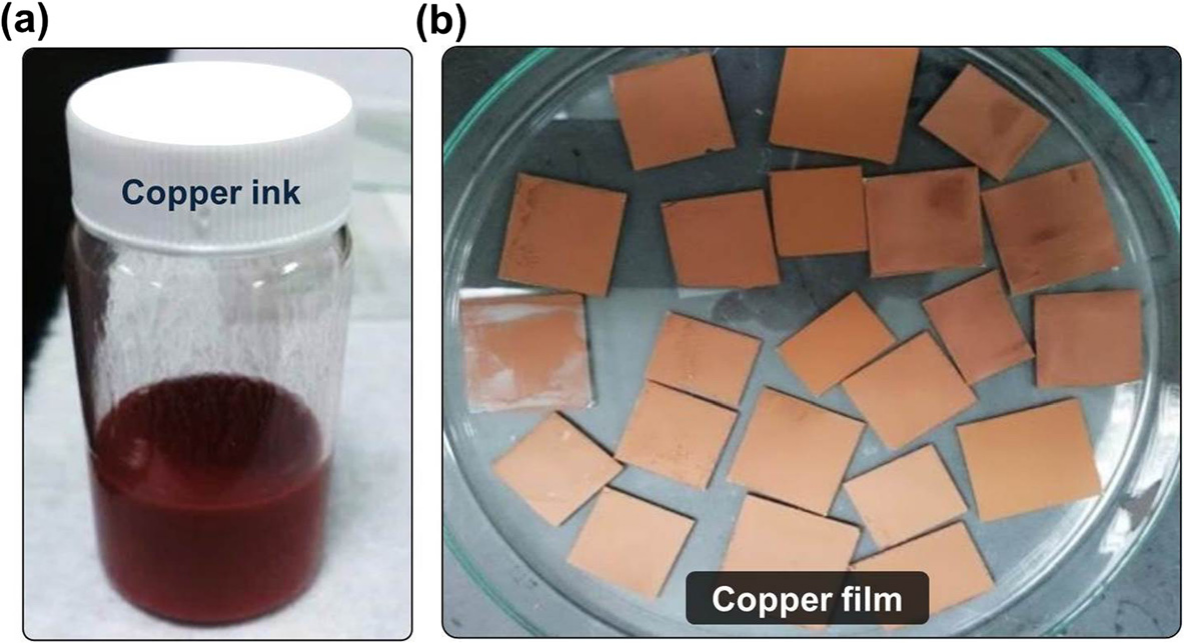
Photograph of **a** fabricated conductive copper ink with the solvent proportion of 1:2 and **b** calcined copper film coated on the glass substrate

After preparing the copper ink, the conductive copper film applied on the glass substrate can be obtained using the copper ink with a calcination process. Figure 2 shows a top-view scanning electron microscope (SEM) image of the fabricated copper nanoparticles with an average diameter of 400~500 nm. Figure 3a shows the photograph of the fabricated conductive copper ink with the solvent proportion of 1:2 (water-free alcohol/tert-butanol). And copper films coated on the glass substrates were calcined at 250 °C in nitrogen atmosphere for 30 min, which is shown in Fig. 3b. Sheet resistances of copper films were measured by using the four-point probe method. To optimize the sheet resistance of the copper film, the solvent proportion for copper ink is adjusted systematically. Table 1 shows the sheet resistance of the copper films with solvent proportions of 1:4, 1:2, and 1:1. We find the lowest sheet resistance of 47.5 mΩ/□ of the copper film for the solvent proportion of 1:2. In our experiment, the solvent tert-butanol plays a critical role in preventing particle agglomeration and creating well-dispersed nanoparticles. Importantly, the concentration of tert-butanol has a significant influence on dispersion quality. We speculated that the copper nanoparticles could generate the agglomeration phenomenon under the solvent proportion of 1:1 since the concentration of tert-butanol in the solution is not enough to disperse the copper nanoparticles. Oppositely, highly dispersed copper nanoparticles could be conjectured for the higher solvent proportion of 1:4. Both solvent proportions (1:1 and 1:4) would produce bad film compactness and non-uniform film thickness, which caused the higher sheet resistance. Relatively, the solvent proportion of 1:2 is the appropriate parameter because of moderate dispersion for copper nanoparticles. This resulted in a uniform copper film with a lower sheet resistance. Thus, the ratio of the weight percentage of water-free alcohol to that of tert-butanol is preferably 1:2.

**Table 1:** The sheet resistances of copper films with different solvent proportions of copper inks

Substrate	Solvent proportion (water-free alcohol/tert-butanol)	Sheet resistance (mΩ/□)
Glass	1:4	183
Glass	1:2	47.5
Glass	1:1	97.2

### Calcination Parameters

Additionally, in order to further investigate the influence of calcination parameters on the sheet resistance of copper films, the calcination time and its temperature are varied. The sheet resistances of copper inks with the solvent proportion of 1:2 at different calcination temperatures and times are listed in Table 2. Obviously, the sheet resistance of the copper film is gradually increased from 7.4 to 47.6 mΩ/□ as calcination temperature is decreased from 400 to 250 °C. In addition, we can see that the copper film shows no conductivity at a calcination temperature of 200 °C, because copper nanoparticles were melted slightly and cannot form the connections when the calcination temperature is 200 °C or at even lower temperatures. Naturally, if the copper film is not calcined, its sheet resistance cannot be measured. For the calcination time, the sheet resistance of the copper film can be improved by increasing the time. The sheet resistance is reduced from 41.3 to 40.6 mΩ/□ when the calcination time is increased from 30 to 60 min. This slight variation of sheet resistance indicates that the fabrication time can be shortened. Significantly, the low-temperature process (<300 °C) of the copper ink is required for practical applications such as HIT solar cells and PCBs. Therefore, we majorly focused on the electrical properties of copper films at a calcination temperature of 250 °C. A low sheet resistance of 47.6 mΩ/□ is obtained at that calcination temperature. Such low-temperature calcination is of vital importance for the semiconductor manufacturing industry. Furthermore, according to the thickness (8 μm) and sheet resistance (47.6 mΩ/□) of our copper film, a resistivity of 3.8 × 10^−5^ Ω cm for this copper film can be calculated. This value is much lower than the previously reported ones ranging from 1.67 × 10^−3^ to 2.46 × 10^−3^ Ω cm for copper paste [22] and is also lower than that of commercial silver paste (2 × 10^−4^ Ω cm) made by Jujo Chemical Co., Ltd. Furthermore, this order is also comparable to that of copper pastes applied in silicon solar cells [23]. These observations strongly show that our copper ink has great potential for the requirements of the industry.

**Table 2:** The sheet resistances of copper films with different calcination parameters

Calcination temperature (°C)	Time (min)	Sheet resistance (mΩ/□)
400	30	7.4
350	30	27.2
300	30	41.3
300	60	40.6
250	30	47.6
250	60	45.4
200	30	–
Non-calcination	0	–

### Morphology

**Fig. 4:**
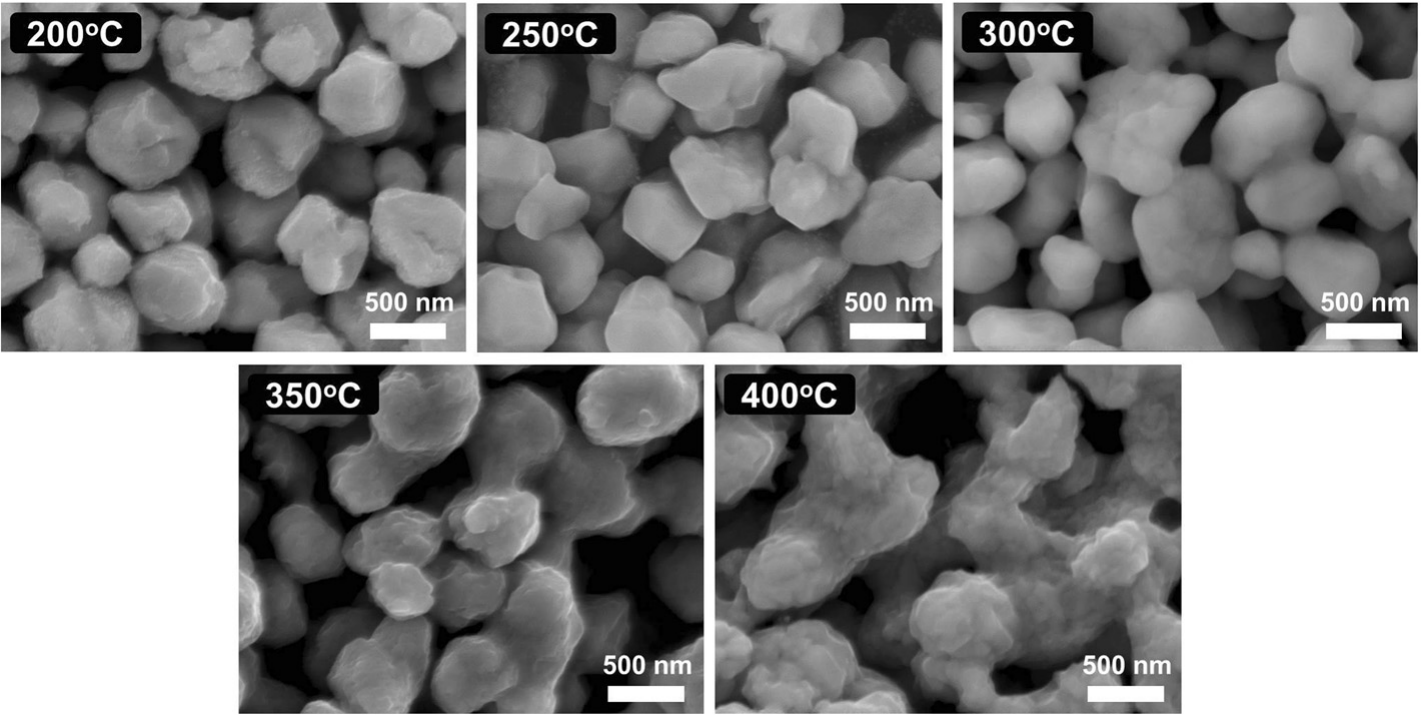
Top-view SEM images of copper films with different calcination temperatures ranging from 200 to 400 °C for 30 min

To further investigate the morphological variation of the copper film with different calcination temperatures, a standard SEM is typically used. Figure 4 shows SEM images of the fabricated copper films with different calcination temperatures within a range of 200–400 °C for 30 min. We can see that copper nanoparticles of the calcined films are apparently melted and connected with the surrounding nanoparticles as the calcination temperature rises gradually from 200 to 400 °C. This can facilitate and enhance the electron transmission between copper nanoparticles. Therefore, the calcination temperature has a great influence on the electrical properties of the copper film. Certainly, copper nanoparticles of copper films with a calcination temperature of 300 °C for 60 min must be also melted and connected. That is why the sheet resistance of the copper film is slightly changed when the calcination time is increased from 30 to 60 min.

### Antioxidative Stability

**Fig. 5:**
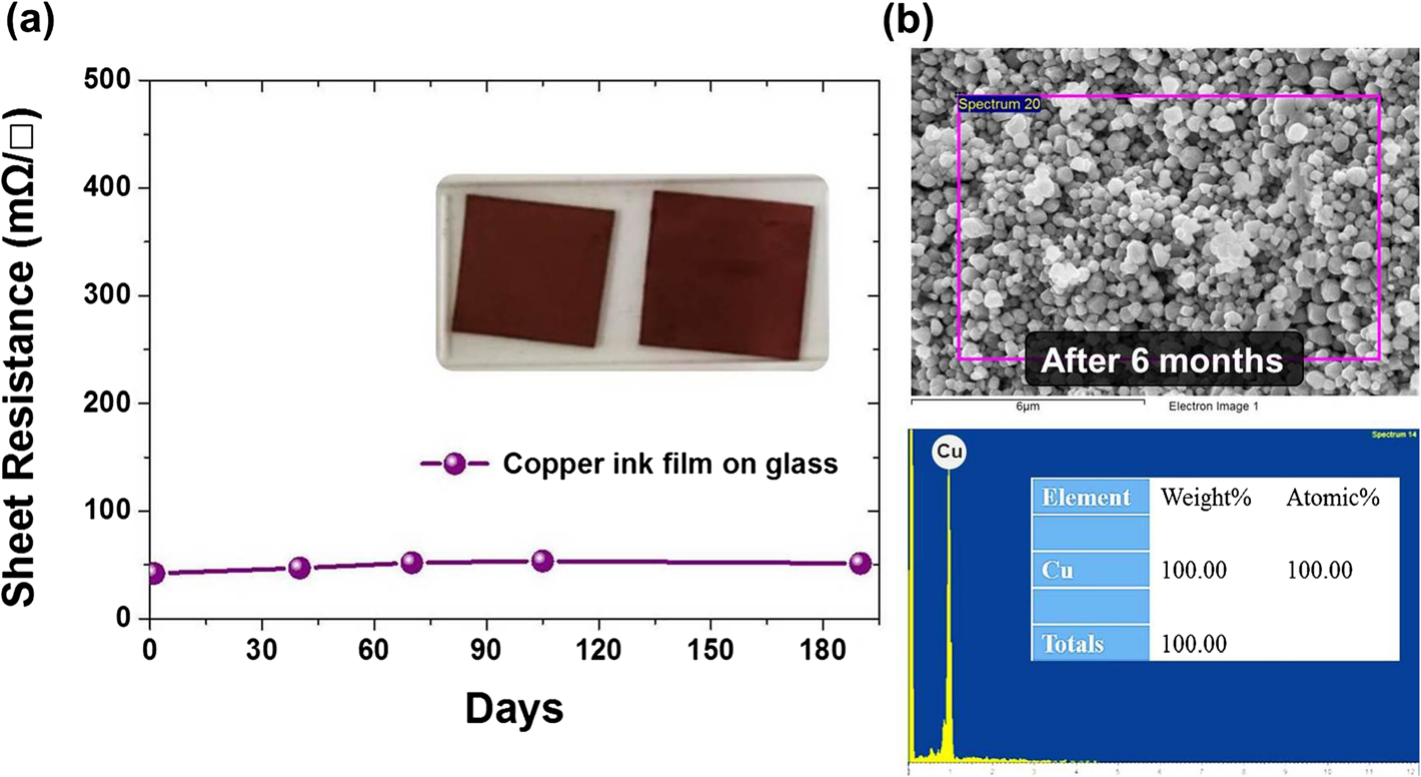
**a** The long-term antioxidation stability testing of antioxidative conductive copper ink. **b** EDS spectrum obtained for the copper film placed in an atmospheric environment for more than 6 months

In general, copper nanoparticles are easy to oxidize in ambient conditions. That is the main obstacle for using conductive copper ink because the appearance of copper oxide will result in decreasing the electrical conductivity. Thus, preventing copper nanoparticles from oxidization becomes an important issue. For this reason, the stability and resistance to oxidation of the conductive copper inks during the storage period were also observed. Figure 5a shows the measured sheet resistance of our copper film as a function of time. We can see that the sheet resistance of the copper film with a calcination of 250 °C was increased from 47.6 to 50.1 mΩ/□ with time. It indicates that these highly conductive copper films can be placed in an atmospheric environment for more than 6 months without the oxidation phenomenon. Such merits of good oxidation resistance and long-term stability for our copper films are associated with the polymer protectant utilized during the preparation process of copper nanoparticles. This polymer protectant can protect copper nanoparticles from oxidation in the air since it would attach to copper nanoparticles and form a protective coating on the surface of the copper nanoparticle, which can reduce exposure to air efficiently and improve long-term storage stability. Moreover, to identify and characterize the elemental composition of copper films after storing for a long period of time, EDS was utilized. A typical EDS spectrum taken on the copper film is shown in Fig. 5b. We can see that the quantitative EDS analysis does not show any oxygen elements for our copper film even if it was placed in an atmospheric environment for more than 6 months. These results indicate that our copper films have high antioxidant capacity and excellent stability in storage time. Accordingly, these antioxidative conductive copper inks are applicable to the field of printable electronic devices [24–28]. For example, the copper ink can be printed or injected to form the PCB or RFID in electronic products. Furthermore, thanks to the property of the oxidation-resistant conductive copper ink, the resistance of the ink or the formed electrodes can be maintained for a long time, which advances the lifetime and stability of various printable electronic products. Therefore, our study for preparing antioxidative conductive copper ink is undoubtedly very practical and offers the advantages of low cost and high quality for electronics manufacturing.

## Conclusions

In summary, we have provided a method for preparing antioxidant conductive copper ink and investigate the characteristics of copper films with different solvent proportions and calcination parameters. The ratio of the weight percentage of water-free alcohol to that of tert-butanol is preferably 1:2. For electrical resistivity of the copper film, a low sheet resistance of 47.6 mΩ/□ is achieved after calcining the copper ink at 250 °C in nitrogen atmosphere for 30 min. In addition, we experimentally demonstrate that the electrical conductivity of copper films can be improved by increasing the calcination temperature. Significantly, our conductive copper film can be placed at room temperature for more than 6 months without the oxidation phenomenon. These unique features of our antioxidative conductive copper ink are particularly useful to many printed electronics applications such as flexible display systems, sensors, photovoltaic cells, and radio frequency identification.
